# ABO blood group and ovarian reserve: a meta-analysis and systematic review

**DOI:** 10.18632/oncotarget.15759

**Published:** 2017-02-27

**Authors:** Jie Deng, Mengmeng Jia, Xiaolin Cheng, Zhen Yan, Dongmei Fan, Xiaoyu Tian

**Affiliations:** ^1^ Department of Obstetrics and Gynecology, The First Affiliated Hospital, and College of Clinical Medicine of Henan University of Science and Technology, Luoyang, China

**Keywords:** ABO blood group, ovarian reserve, systematic review, meta-analysis

## Abstract

Ovarian reserve reflects a woman's fertility potential. The ABO blood group system is a very stable genetic marker. Although many recent studies have explored the relationship between ABO blood group and ovarian reserve, a consensus has not yet been reached. This paper is the first meta-analysis and systematic review of the relationship between ABO blood type and ovarian reserve. We analyzed seven cross-sectional studies evaluating follicle stimulating hormone (FSH) or anti-Mullerian hormone (AMH) levels in 55,113 participants to determine ovarian reserve. This study found no relationship between ABO blood type and ovarian reserve when FSH was used as an indicator of ovarian reserve (A *vs* non-A:OR=1.03, 95% CI:0.96-1.11; B *vs* non-B: OR=0.98, 95% CI:0.75-1.29; AB *vs* non-AB:OR=0.96, 95% CI:0.71-1.30; O *vs* non-O:OR=1.03, 95%CI:0.74-1.43).There was also no relationship between ABO blood type and ovarian reserve when AMH was used as an indicator (A *vs* non-A:OR=0.89, 95% CI:0.76-1.03; B *vs* non-B:OR=1.02, 95% CI:0.80-1.30; AB *vs* non-AB:OR=1.14, 95% CI:0.80-1.64, O *vs* non-O:OR=1.07, 95% CI:0.86-1.34). Overall, the current study found no relationship between ABO blood group and ovarian reserve. However, additional rigorous, high-quality and multi-indicator studies with large sample sizes are required for further verification.

## INTRODUCTION

Ovarian reserve refers to reproductive potential as a function of the number and quality of remaining oocytes [1]. Recently, changes in the social environment, lifestyle and prolonged female reproductive years have led to gradual increases in female infertility [2, 3]. Statistically, approximately 10% of infertile women have decreased ovarian reserve (DOR) [4], causing altered hormone levels that largely clinically manifest as peri-menopausal symptoms, such as hot flashes, sweating, anxiety, irritability, insomnia and other symptoms [5]. DOR also gradually evolves into premature ovarian failure (POF), which contributes to osteoporosis, cardiovascular disease and other related health issues [6].These conditions have serious impacts on a woman's reproductive health and quality of life and also place a certain burden on the family and society.

In evaluating ovarian reserve, recent studies have focused on age, antral follicle count(AFC), and hormones, particularly follicle stimulating hormone(FSH) and anti-Mullerian hormone(AMH), all of which can serve as indicators of ovarian reserve [7]. The basal FSH value, defined as the serum level during the first 2-3 days of the menstrual cycle, can be used for screening, counseling and other diagnostic purposes. The method used for detection is simple, economical, highly reproducible, and widely applied in clinical practice [8].The sensitivity and specificity of AMH in predicting ovarian response are high [9], and this hormone shows no significant fluctuations during the menstrual cycle [10]. AMH measurement is also convenient and rapid, with great potential in clinical application.

ABO blood group antigens are the glycoproteins or glycolipids that are distributed on the red blood cell membrane. These molecules are expressed not only on the surface of red blood cells but also on a variety of human cells and tissues, including epithelial cells, platelets, the vascular endothelium and neurons [11, 12]. Studies have shown that ABO blood group is related to the occurrence of cardiovascular disease, cancer and other diseases [13, 14].In initial studies of the pathogenesis of ovarian hyperstimulation syndrome (OHSS), it was found that von Willebrand factor (VWF) levels might correlate with the development of OHSS [15]. Donnell [16] found that OHSS occurs when compared with patients with blood type A, blood type O in patients with VWF and VIII factor concentration will decrease. In addition, Binder *et al* [17] found that patients with blood type O develop OHSS less frequently, while blood type A is more likely to be associated with early-onset OHSS. Such findings promoted interest in the relationship between ABO blood type and DOR. Although recent studies have explored the relationship between blood type and ovarian reserve, the results are contradictory. In 2011, Nejat *et al* [18] suggested that blood type O was a risk factor for DOR, whereas presence of the A antigen (blood types A or AB)was protective for ovarian reserve. In 2013, Timberlake *et al* [19] failed to find a link between blood type and reduced ovarian reserve. Furthermore, Lin *et al* [20] suggested that the incidence of DOR was low in Chinese women with blood type O and that presence of the B antigen (blood types B or AB) was a risk factor for DOR. Thus, controversy remains with regard to whether blood type is related to ovarian reserve and which antigen is protective or a risk factor.

To elucidate the relationship between ABO blood type and ovarian reserve, we used literature to date to conduct a meta-analysis and systematic review.

## RESULTS

### Screening results

**Figure 1:**
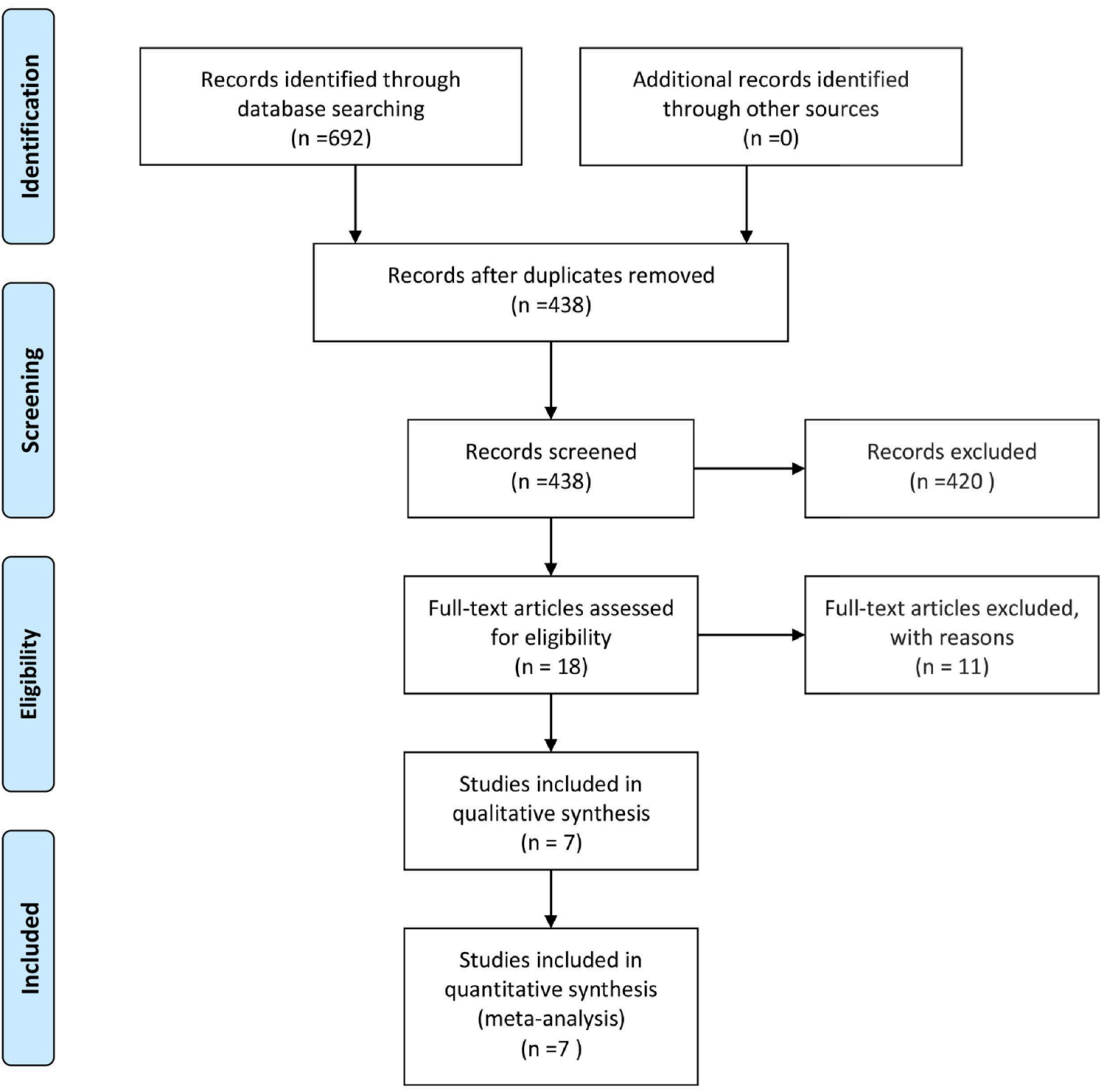
Flow-chart of study selection

As shown in Figure 1, 692 articles were initially identified using a comprehensive search, of which 438 remained after duplicate results were removed. Among these studies, 420 were deemed irrelevant after reading the abstracts and/or did not meet the inclusion criteria; thus, they were excluded. A total of 18 potentially eligible articles were reviewed by analyzing the full text. Based on this analysis, seven published studies [18–24] were included in this meta-analysis.

### Study characteristics

The selected studies were conducted in four countries and published between 2011 and 2016. A total of 55,113 participants were included, with 8200 in the decreased ovarian reserve group and 46,913 in the control group. The studies were divided into two aspects according to the method used to determine ovarian reserve: five used FSH, and two used AMH. The major characteristics of the included studies are shown in Table 1.

**Table 1:** Characteristics of the included studies

	Determination of ovarian reserve	Age(year	Race	case	control	Main result
Nejat 2011 [18]	FSH	<45 (median age35, inter-quartile range 30–39)	American (different ethnicities)	65 A 16 (24.6%) B 8 (12.3%) AB 1(1.5%) O 40 (61.5%)	479 A 173(36.1%) B 77(16.0%) AB 24(5.0%) O 205(42.8%)	A antigen (blood types A or AB) was protective for ovarian reserve, blood type O was a risk factor for DOR.
Timberlake 2013 [19]	FSH	Average age 36.5(Range27-46)	American (different ethnicities)	151 A 68(45.0%) O 64(42.4%)	154 A 62(40.2%) O 71(46.1%)	Found no relationship between ABO blood group and ovarian reserve.
Lin 2014 [20]	FSH	<45 average age 32.47±4.58	China	3356 A 955(28.5%) B 1212(36.1%) AB 395(11.8%) O 794(23.6%)	32123 A 8906(27.7%) B 10183(31.7%) AB 3245(10.0%) O 9789(30.5%)	Blood type O was protective for ovarian reserve; B antigen (blood types B or AB)was a risk factor for DOR.
Sengul 2014[21]	FSH	Range 18-45	Turkey	62 A 28(45.1%) B 8(12.9%) AB 4(6.5%) O 22(35.5%)	438 A 171(39.0%) B 69(15.8%) AB 43(9.8%) O 155(35.4%)	Found no relationship between ABO blood group and ovarian reserve.
Mu 2016[22]	FSH	average age 31.1±4.37	China	2589 A 814(31.4%) B 590(22.8%) AB 175(6.8%) O 1010(39.0%)	12286 A 3794(30.9%) B 3061(24.9%) AB 951(7.7%) O 4480(36.5%)	B antigen (blood types B or AB)was protective for ovarian reserve; blood type O was a risk factor for DOR.
Mouzon 2012[23]	AMH	average age 35.6±5.1	France	277 A 113(40.8%) B 40(14.4%) AB 11(3.9%) O 113(40.8%)	739 A 311(42.0%) B 89(12.0%) AB 25(3.4%) O 314(42.5%)	Found no relationship between ABO blood group and ovarian reserve.
Pereira 2013[24]	AMH	Blood type A average age37.1±4.8; blood type B average age 37.0±4.6; blood type AB average age 37.3±4.7; blood type O average age 37.7±4.7	American	1700 A 611(35.9%) B 303(17.8%) AB 88(5.1%) O 698(41.0%)	694 A 274(39.5%) B 130(18.7%) AB 32(4.6%) O 258(37.2%)	Found no relationship between ABO blood group and ovarian reserve.

FSH: follicle stimulating hormone

AMH: anti-Mullerian hormone

DOR: decreased ovarian reserve

### Quality assessment

The results of the quality assessment are shown in Table 2, with the scores ranging from 6 to 8. All of the studies were identified as having relatively high quality.

**Table 2:** Newcastle-Ottawa Scale table

	the selection of the study groups	he comparability of the groups	the ascertainment of exposure or outcome of interest	Total
Nejat 2011	3	1	3	7
Timberlake 2013	3	2	3	8
Lin 2014	3	2	3	8
Sengul 2014	3	2	3	8
Mu 2016	3	2	3	8
Mouzon 2012	3	0	3	6
Pereira 2013	3	0	3	6

### Outcomes

**Figure 2:**
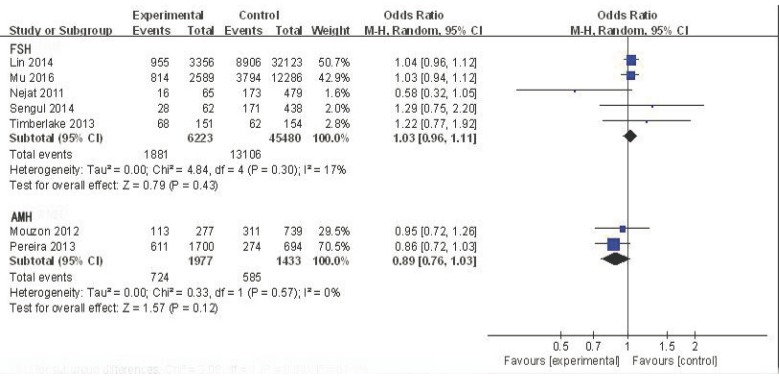
Forest plot of blood group A

**Figure 3:**
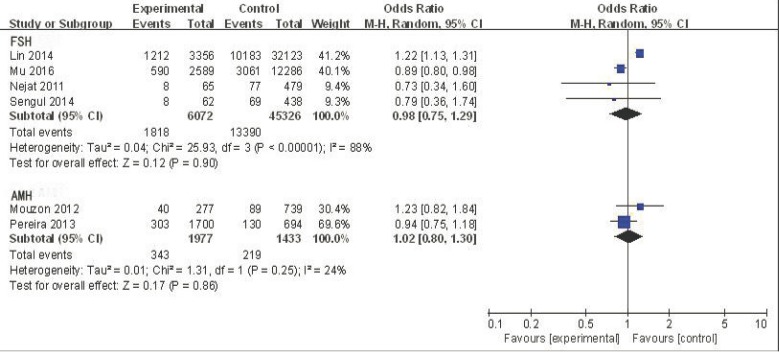
Forest plot of blood group B

**Figure 4:**
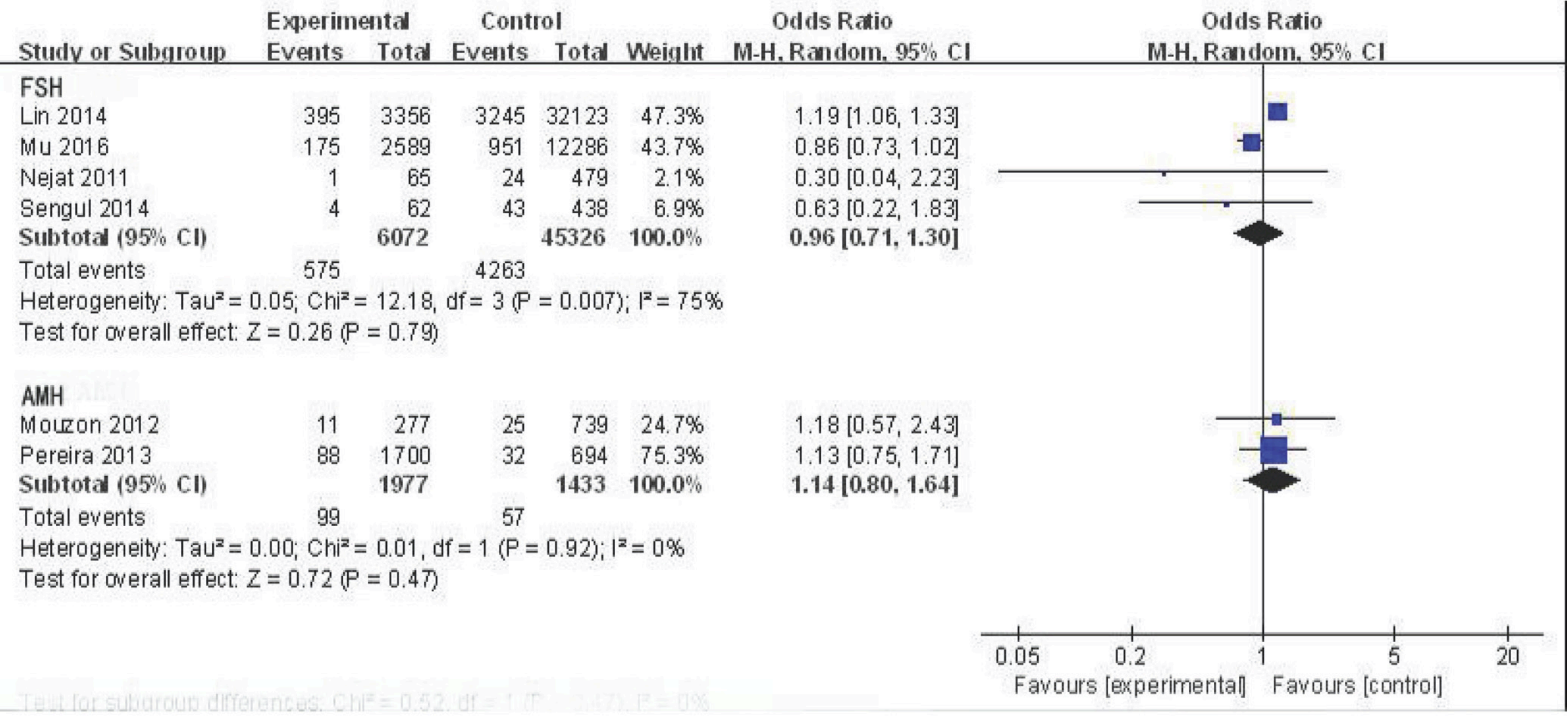
Forest plot of blood group AB

**Figure 5:**
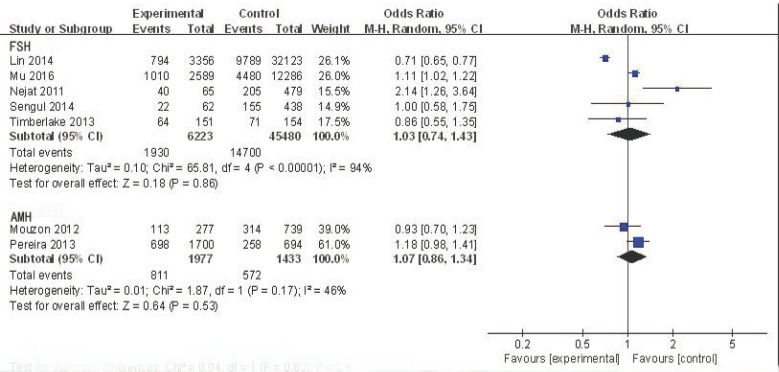
Forest plot of blood group O

The five studies that used FSH to detect ovarian reserve were analyzed to determine the odds ratio (OR) value of decreased ovarian reserve in the ABO blood group. For blood type A, the effective value was1.03, and the 95% confidence interval (CI) was 0.96-1.11 (Figure 2). This finding suggests no association between blood type A and ovarian reserve in this sample with low heterogeneity (I^2^ = 17%). Although not all studies on blood type B and AB supply data have also found similar results (OR = 0.98, 95% CI:0.75-1.29; OR = 0.96, 95% CI:0.71-1.30, respectively) (Figures 3 and 4), there was no association between blood type B or blood type AB and ovarian reserve in these samples with high heterogeneity(I^2^ = 88% and I^2^ = 75%, respectively) (Figure 5). Similarly, there was no association between blood type O and ovarian reserve (OR = 1.03, 95% CI: 0.74-1.43), and this sample also exhibited high heterogeneity (I^2^ = 94%). These data indicate that there is no relationship between ABO blood type and ovarian reserve when FSH is used as the indicator of ovarian reserve.

The two studies that employed AMH to detect ovarian reserve were also analyzed. For blood type A, the OR was 0.89, and the 95% CI was 0.76-1.03 (Figure 2), suggesting no association between blood type A and ovarian reserve in this sample with low heterogeneity (I^2^ = 0%). Similarly, no association was found between blood type B and ovarian reserve (OR = 1.02, 95% CI: 0.80-1.30; Figure 3); this sample also exhibited low heterogeneity (I^2^ = 24%). A similar result was found for blood type AB (OR = 1.14, 95% CI: 0.80-1.64; Figure 4), suggesting no association between blood type AB and ovarian reserve, low heterogeneity was also observed in this sample(I^2^ = 0%). The OR (1.07) and 95% CI (0.86-1.34, Figure 5) for blood type O also suggested no association between blood type O and ovarian reserve in this sample with low heterogeneity (I^2^ = 46%). The above data show that there is no relationship between ABO blood type and ovarian reserve when AMH is used as the indicator of ovarian reserve.

In conclusion, the current studies failed to find an association between ABO blood group and ovarian reserve.

### Sensitivity analyses

Sensitivity analysis of the study with high heterogeneity (blood type B, blood type AB, blood type O study) found that after exclusion of the Lin *et al* [20] study, the heterogeneity for blood type B and blood type AB was reduced to 0 (I^2^ = 0%), and that for blood type O also decreased significantly (I^2^ = 58%). After exclusion of the Lin *et al* study, the effective OR for blood type B was 0.89 (95% CI:0.8-0.98), changing the conclusion and indicating that blood type B is a protective factor for ovarian reserve. This sensitivity analysis showed that the results were not stable, and the high heterogeneity was derived from the Lin *et al* study. Detailed analysis of the Lin *et al* study with regard to the inclusion and exclusion criteria, research design, sample size, quality evaluation and other characteristics identified no differences in the inclusion and exclusion criteria, research design, quality evaluation or other characteristics relative to the other studies. In contrast, the sample size in the Lin *et al* study was twice as large as the sum of the other four studies, which influenced the outcome of the meta-analysis.

## DISCUSSION

In recent years, several biological studies have indicated that blood type and ovarian reserve may be inextricably linked. The direct translation product of the ABO blood group gene is glycosyltransferase. FSH and luteinizing hormone (LH) receptors are heavily glycosylated proteins, and FSH receptor expression in ovarian granulosa cells functions together with activated LH receptors to promote follicular development [25].Dharmesh and Baenziger [26] found that glycosyltransferases maintain the terminal glycosylation of LH with galactose-4-SO4 oligosaccharides, thereby affecting the cycle half-life of LH. Thus, it is reasonable to speculate that glycosyltransferases may be, to some extent, related to gamete production [27] and that a lack of these enzymes may lead to impaired gamete formation. In addition, studies have found that the NR5A1 gene is located at the core of the ABO gene and plays a role in transcriptional regulation in the hypothalamic-pituitary-ovary (H-P-O) axis [28].Blocking the transcription and translation of the NR5A1 gene in mice causes different degrees of developmental defects in newborn gonads and adrenal glands, disturbed FSH and LH secretion by the adenohypophysis, and an abnormal hypothalamic median eminence structure [29]. A study of human ovarian tissue found that NR5A1 is expressed during almost every stage of a woman's life: the earliest expression was found in the fetus, with expression continuing to adulthood [30]. When NR5A1 expression is dysregulated, the follicles in the ovary cortex cannot mature normally, and the ovarian tissue cannot support normal ovulation.

There is no clear evidence from biological studies for a direct link between blood type and ovarian reserve, and recent clinical studies have failed to reach a consensus regarding the relationship between the two, making systematic review particularly important. This paper reports the first meta-analysis and systematic evaluation of the relationship between ABO blood type and ovarian reserve. Our systematic review included seven studies that investigated FSH and AMH, two indicators of ovarian reserve, with a total of 55,113 participants.

The current study failed to find an association between ABO blood group and ovarian reserve. This conclusion is not consistent with some clinical studies, and this discrepancy may exist because the Nejat *et al* [18] study did not establish a set time in the menstrual cycle at which to obtain serum samples (it should be noted that in the other four studies using FSH, the level was measured on the third day of the menstrual cycle). Although the study attempted to reduce the impact by analyzing E2 levels concomitantly with FSH, this may have potentially introduced bias. In addition, the authors used data from two centers to reduce race effects, but this may have increased other confounding factors. Accumulating evidence suggests that race and ethnicity influence the frequency of different blood types. For example, the frequencies for the O, A, B, and AB blood types are 44%, 42%, 10%, and 4%, respectively, in the United States but are 29%, 27%, 32%, and 13% [31], respectively, in China. In addition, there is evidence that ovarian reserve may also differ dramatically among races: compared with white women, Latina and Chinese women may have lower ovarian reserve and be at increased risk of early menopause [32]. The main confounding factors affecting ovarian function, such as smoking, previous chemotherapy or radiotherapy, ovarian surgery, and endometriosis [33], were not fully considered in the study design and may have also increased the risk of outcome bias.

Interestingly, a 2014 study by Spitzer *et al*. [34] did not use a direct serological method to determine ovarian reserve but rather utilized mature oocytes, fertilization rates and pregnancy rates to predict ovarian reserve. Their conclusion was similar to ours, finding no relationship between ABO blood group and ovarian reserve.

There are several limitations of this study. First, analysis of previous studies revealed that heterogeneity cannot be ignored. Although the selected articles did not differ with regard to inclusion and exclusion criteria, research design, quality assessment and other aspects, the large sample size of the Lin *et al* study made the results unreliable. Second, although AMH is a good prognostic marker of ovarian reserve, only a few recent studies have employed AMH as an indicator. Finally, our study included only infertile women, limiting the applicability of our findings to the general population.

In summary, the clinical evidence generated thus far has not identified a link between ABO blood type and ovarian reserve. However, the biological research hypotheses about the relationship between ABO blood type and ovarian reserve and the conclusion of this paper differ; therefore, it is necessary to confirm these results with additional far-reaching biological studies. Moreover, many different indices are currently used to measure ovarian reserve, and additional multi-indicator studies are needed in the future. In addition, considering the heterogeneity of our analysis and the limited number of studies, our results require further verification with additional rigorous, high-quality studies with large sample sizes.

## MATERIALS AND METHODS

The protocol of this review was registered in PROSPERO under registration number CRD 42016046330 (http://www.crd.york.ac.uk/PROSPERO/).

### Inclusion and exclusion criteria

The inclusion criteria were as follows, 1) The case group and control group needed to be from the same period and region; 2) FSH or AMH was evaluated to indicate ovarian reserve in the case and control groups (FSH > 10 IU/L or AMH < 1.5 pg/ml was considered DOR [35]); 3) ABO blood group typing was clear, and the data were extractable; 4) Sufficient data were available to calculate the OR and 95% CI.

The following types of studies were excluded: non-original studies; only abstracts without full-text data; repeated reports containing similar information or poor quality data that could not be used.

### Search strategy

Electronic databases, including PubMed, the Science Citation Index (SCI), Cochrane Central, EMBASE, CINAHL, and Web of Science were searched. The search cut-off date was August 1, 2016.Both medical subject heading (MeSH) and text-word searches were employed using the following terms: “blood type” and “ovarian reserve”. All the references included in this article were also searched to identify eligible studies. The language was restricted to English, but there were no country restrictions.

### Data collection

Studies were independently selected by two reviewers based on the inclusion and exclusion criteria. Information regarding the literature, including the authors’ names, institutions, or journals, was extracted by the reviewers during the selection process. Disagreements were resolved through discussion; if no consensus was reached, a third reviewer was consulted. If two or more reports included the same participants, then only the most recent report was included in the meta-analysis.

### Statistical analyses

The main outcome was the incidence of DOR. Statistical analyses were performed using RevMan version 5.3, and ORs and 95% CIs were used to measure outcomes. Q and Higgins I^2^ statistics were used to examine heterogeneity among studies. When *P* ≥ 0.1 and I^2^≤50%, the included studies were considered to have little heterogeneity; when *P* < 0.1 and I^2^ > 50%, the included studies were considered to have substantial heterogeneity [36].Because the random-effects model is relatively conservative, it was adopted to make the results more reliable [37]. Sensitivity analyses were performed to assess the impact of each study on the overall analysis to identify the source of heterogeneity.

### Quality assessment

The Newcastle-Ottawa Scale [38] was used to evaluate the research from three aspects: (1) the selection of the study groups; (2) the comparability of the groups; and (3) the ascertainment of exposure or outcome of interest. Each study could receive a score from 0 points (the lowest quality) to 9 points (the highest quality). High-quality studies were defined as those with a score of more than 6 points; otherwise, the studies were rated as poor quality.
